# Atlanta Medical College–Close of the Session

**Published:** 1859-09

**Authors:** 


ATLANTA MEDICAL COLLEGE—CLOSE OF THE SESSION.

The Course of Lectures in the Atlanta Medical College closes with the issue of this number of the Journal, and we have no hesitation in asserting, that the Session has been an eminently successful one. The increase in the number of Students has been, over any previous year, about one-fourth, the Matriculating list of 1859 numbering 166, and representing Virginia, North Carolina, South Carolina, Florida, Georgia, Alabama, Mississippi, Arkansas and Texas, and extravagant though it may be considered, we should not do justice to those composing it, if we did not say, that in moral character, correct deportment and devotion to study, we doubt whether an equal class has ever assembled in the walls of a Medical College. We are much pleased, also, to note the fact, that there is a marked and progressive elevation, year after year, strikingly exhibited in the preliminary educational preparation of those who attend here for medical instruction.

This Institution is now furnishing Students with an opportunity for attending two Courses of College instruction annually—one a preparatory or elementary, and the other more practical—and it is absolutely certain that the great advantage of this plan is already apparent in the greater proficiency of those who have been in connection with the Institution during the entire Annual Term just ended. As will be perceived, by reference to the advertisement upon the cover, this increase of facilities involves no additional expense to the Student, but is only undertaken as an inducement for more thorough preparation upon the part of those seeking a place in the Medical profession, before entering upon its active and responsible duties, and in addition, is intended to be regarded as another manifestation of the continuance of the *progressive* character which has been so strikingly manifested in the history of the Institution.

Without intending to indulge in any spirit of boasting, or to make any invidious or unfair comparisons with other Medical Colleges, less fortunately situated, we cannot forbear to direct attention to the fact that we are located more favorably for the extension of the term of medical instruction to something like the time occupied annually in our literary institutions (which we regard as holding out the best prospect for the improvement of medical education, and the elevation of the standard of qualification,) than any other Medical College in the South, and as already intimated, the Faculty of this Institution, in a spirit of self sacrifice, and an earnest desire to contribute to the more thorough qualification of those who enter the profession by their sanction, have taken advantage of their favorable position, and are already able to demonstrate the beneficial results of this important improvement.

